# EBV-Encoded LMP1 Upregulates Ig*κ* 3′Enhancer Activity and Ig*κ* Expression in Nasopharyngeal Cancer Cells by Activating the Ets-1 through ERKs Signaling

**DOI:** 10.1371/journal.pone.0032624

**Published:** 2012-03-01

**Authors:** Haidan Liu, Zhi Duan, Hui Zheng, Duosha Hu, Ming Li, Yongguang Tao, Ann M. Bode, Zigang Dong, Ya Cao

**Affiliations:** 1 Cancer Research Institute, Xiangya School of Medicine, Central South University, Changsha, China; 2 State Key Laboratory of Medical Genetics, Clinical Center for Gene Diagnosis and Therapy, Central South University, The Second Xiangya Hospital, Changsha, China; 3 Department of Cardiothoracic Surgery, Central South University, The Second Xiangya Hospital, Changsha, China; 4 The Hormel Institute, University of Minnesota, Austin, Minnesota, United States of America; Florida International University, United States of America

## Abstract

Accumulating evidence indicates that epithelial cancer cells, including nasopharyngeal carcinoma (NPC) cells, express immunoglobulins (Igs). We previously found that the expression of the kappa light chain protein in NPC cells can be upregulated by the EBV-encoded latent membrane protein 1 (LMP1). In the present study, we used NPC cell lines as models and found that LMP1-augmented kappa production corresponds with elevations in ERKs phosphorylation. PD98059 attenuates LMP1-induced ERKs phosphorylation resulting in decreased expression of the kappa light chain. ERK-specific small interfering RNA blunts LMP1-induced *kappa light chain* gene expression. Luciferase reporter assays demonstrate that immunoglobulin *κ* 3′ enhancer (3′E_κ_) is active in Igκ-expressing NPC cells and LMP1 upregulates the activity of 3′E_κ_ in NPC cells. Moreover, mutation analysis of the PU binding site in 3′E_κ_ and inhibition of the MEK/ERKs pathway by PD98059 indicate that the PU site is functional and LMP1-enhanced 3′E_κ_ activity is partly regulated by this site. PD98059 treatment also leads to a concentration-dependent inhibition of LMP1-induced Ets-1 expression and phosphorylation, which corresponds with a dose-dependent attenuation of LMP1-induced ERK phosphorylation and kappa light chain expression. Suppression of endogenous *Ets-1* by small interfering RNA is accompanied by a decrease of Ig kappa light chain expression. Gel shift assays using nuclear extracts of NPC cells indicate that the transcription factor Ets-1 is recruited by LMP1 to the PU motif within 3′E_κ_ *in vitro*. ChIP assays further demonstrate Ets-1 binding to the PU motif of 3′E_κ_ in cells. These results suggest that LMP1 upregulates 3′E_κ_ activity and *kappa* gene expression by activating the Ets-1 transcription factor through the ERKs signaling pathway. Our studies provide evidence for a novel regulatory mechanism of kappa expression, by which virus-encoded proteins activate the *kappa* 3′ enhancer through activating transcription factors in non-B epithelial cancer cells.

## Introduction

The restriction of immunoglobulin (Ig) expression to cells of the B-cell lineage is well established. However, we found Ig kappa light chain was “unexpectedly” expressed in epithelial cancer cell lines and epithelial tissues [1], [2], [3]. The expression of Ig kappa light chain in non-hematopoietic tumor cell lines was also reported by other laboratories [4], [5], [6], [7].

Immunoglobulin *kappa light chain* gene expression is under the control of distinct cis-regulatory elements, including promoters and enhancers. Two important *kappa* enhancers: the intronic enhancer (iE_κ_), which lies between the J_κ_-C_κ_ region, and the 3′ enhancer (3′E_κ_), which is located downstream of the C_κ_ region, have been identified [8], [9], [10]. Both enhancers are inactive at the pro-B and pre-B cell stages and active at the Igκ-expressing mature B cell and plasma cell stages [10], [11]. The activity of these enhancers is generally transcriptionally silent in other cells that cannot produce the kappa chain, such as T-lymphoid cells (Jurkat) [10], epithelial cells (HeLa) [10] and NIH3T3 fibroblasts [12]. Based on these observations, the activation of these regulatory elements is generally believed to be required for *immunoglobulin kappa* gene expression and is a B cell lineage-restricted event [10]. Intriguingly, we have found that human iE_κ_ is active in Igκ-expressing nasopharyngeal carcinoma (NPC) cell lines, which is important for kappa light chain expression in these cells [13]. However, whether the other *kappa* enhancer, 3′E_κ_, is functional in Igκ-expressing epithelial cancer cells remains unknown.

The function of enhancers is mediated by DNA binding proteins that are recruited to the regulatory elements of the enhancers. Several positive regulatory elements have been identified in 3′E_κ_, including a consensus PU motif (TTTGGGGAA) for transcription factor Ets-related proteins [10]. The Ets family comprises several subfamilies, including ETS (Ets-1, Ets-2), TCF (Elk-1, Sap-1, etc.), and SPI (PU.1, Spi-B, Spi-C etc.). Family members are identified on the basis of their structural composition and their similarities in the evolutionarily-conserved Ets domains that mediate binding to purine-rich DNA sequences with a central GGAA/T core consensus [14], [15]. Ets family proteins are nuclear proteins and phosphorylation is an important post-translational modification of many Ets family members, which can affect their transcriptional activities and DNA-binding activities [15]. In B cells, binding of the PU.1 protein to the kappa 3′ enhancer play an important role in 3′E_κ_ function [16]. Phosphorylation of PU.1 at Ser148 is required for the interaction of PU.1 with Pip on DNA and this phosphorylation can regulate the transcriptional activity of PU.1 [17]. However, the PU.1 protein is exclusively expressed in hematopoietic cells [15], [18] and is unlikely to execute regulatory function in Igκ-expressing epithelial cancer cells. Recent study by using chromatin immunoprecipitation coupled with genome-wide promoter microarrays to query the occupancy of three ETS proteins in a human T-cell line, revealed that redundant occupancy was frequently detected, while specific occupancy was less likely [19]. Thus, we can speculate that, If 3′E_κ_ is indeed functional in Igκ-expressing epithelial cancer cells, other Ets family proteins are more likely to play a role in 3′E_κ_ activity than PU.1. Therefore, we decided to further investigate that which transcription factor(s) bound to the PU binding site of 3′E_κ_ and whether the binding is important for 3′E_κ_ functional activation in Ig kappa-expressing epithelial cancer cells.

Our previous study showed that the *kappa light chain* gene was expressed in NPC and other epithelial tumor cells. Most interestingly, we found that the levels of the kappa light chain were substantially higher in LMP1-positive cells compared to LMP1-negative cells [2]. Because of its transforming and tumorigenic activities, LMP1 is considered to be a major oncogenic protein encoded by EBV. LMP1 mediates a variety of cellular signaling pathways including NF-κB, c-Jun-NH_2_-terminal kinases (JNKs), p38/MAPK, PI3K/Akt and JAK/STAT and causes transcriptional upregulation of several cellular genes, such as *il-6, il-8, bcl-2, cd23, a20* and *egfr* [20], [21], [22]. LMP1 can also activate the Ras/ERK/MAPK signaling pathway [23], and the Ras/MAPK signaling kinases, Raf, MEK and ERKs, are activated in LMP1-expressing nasopharyngeal epithelial cells [24]. Moreover, Kim [25] reported that stable transfection of the *LMP1* gene into MDCK cells induced expression of Ets-1, suggesting that *Ets* might be a target gene of LMP1. As mentioned above, Ets-1 and Ets-2 are subfamily members of Ets-related proteins. Both of them are nuclear targets of the Ras signaling pathway and phosphorylation of conserved threonine residues, Thr38 and Thr72, is a necessary molecular event for Ras-mediated activation of these transcription factors [26]. Cumulatively, an LMP1/ERK/Ets/kappa signaling cascade might exist by which LMP1 upregulates kappa light chain expression in NPC cells.

In the present study, The MEK inhibitor, PD98059, was used to investigate the role of the ERKs pathway in LMP1-enhanced kappa light chain production in NPC cells. The data presented here demonstrate that LMP1-augmented kappa production corresponds with elevations in ERKs phosphorylation. PD98059 inhibits LMP1-induced ERKs phosphorylation resulting in decreased expression of the kappa light chain. ERK-specific small interfering RNA blunts LMP1-induced *kappa light chain* gene expression. Luciferase reporter assays demonstrate that 3′E_κ_ is active in Igκ-expressing NPC cells and stable LMP1 expression upregulates the activity of 3′E_κ_ in NPC cells. Mutations of the PU binding site on 3′E_κ_ significantly decrease LMP1-enhanced 3′E_κ_ activity. LMP1-induced 3′E_κ_ activity is dramatically inhibited by the ERKs upstream kinase inhibitor, PD98059. Treatment of PD98059 also leads to a concentration-dependent inhibition of LMP1-induced Ets-1 expression and phosphorylation, which corresponds with a dose-dependent attenuation of LMP1-induced ERK phosphorylation and kappa light chain expression. The knockdown of endogenous *Ets-1* by small interfering RNA is accompanied by a decrease of Ig kappa light chain expression. Gel shift assays using nuclear extracts prepared from various NPC cell lines confirm that the transcription factor Ets-1 is recruited by LMP1 to the PU motif of the human *kappa light chain* gene. ChIP assays further demonstrate that Ets-1 directly binds to the PU motif of 3′E_κ_ in cells. These results suggest that LMP1 upregulates 3′E_κ_ activity and *kappa light chain* gene expression by activating the Ets-1 transcription factor through the ERKs signaling pathway.

## Materials and Methods

### Cell lines and cell culture

HNE2 is an EBV-LMP1-negative human NPC cell line. HNE2-LMP1 is a cell line that constitutively expresses LMP1 after the introduction of full-length *LMP1* cDNA into HNE2 cells [27]. The human myeloma cell lines, XG6, which expresses the cytoplasmic λ light chain, and XG7 that expresses the cytoplasmic κ light chain [28], were used as kappa chain negative and positive controls, respectively. Raji is a human B-cell Burkitt's lymphoma cell line. All the cell lines were maintained in RPMI1640 (GIBCO, USA) supplemented with 10% FBS (GIBCO, USA), 1% glutamine, and 1% antibiotics in a 37°C humidified atmosphere containing 5% CO_2_. For the XG6 and XG7 cells, 1 ng/ml rIL-6 (Sigma, St. Louis, MO) was added to RPMI1640 medium supplemented as described above [28]. Cells in logarithmic growth phase were used in all experiments.

### Chemicals and cell treatments

The ERKs upstream kinase MEK inhibitor, PD98059 (Cell Signaling, USA), was prepared as a stock solution of 20 mM in dimethylsulfoxide (DMSO, Sigma). Subconfluent cells were treated with the compound at various concentrations for different times. Detailed treatment procedures are described in the Figure Legends. The final concentration of DMSO in the culture medium was kept at less than 0.1%, which had no significant effect on cell growth. Vehicle controls were prepared for all treatments.

### Plasmids

The human *β-globin* promoter was a 128 bp minimal promoter identical to that used previously [29]. The promoter was obtained by amplification from human HNE2 cellular genomic DNA with the following primers: sense, 5′-*gagctc*acggctgtcatcacttagacctcac-3′, which contains the *Sac I* cloning site; antisense, 5′-*aagctt*taagcaatagatggctctgccctgac-3′, which contains the *Hind III* site. The fragment was inserted into the *Sac I/Hind III* sites of the *pGL3-Basic* vector (Promega, Madison, WI) and the plasmid was designated as *pGL3-β*.

A 313 bp fragment containing the human 3′E_κ_ enhancer core and 90 bp upstream of the enhancer core sequences [10], [30], [31] was cloned. The 3′E_κ_ enhancer fragment was amplified from HNE2 cellular genomic DNA by PCR using specific primers from the human *Ig kappa* gene (GenBank accession no. NG_000834): sense, 5′-*ggatcc*cctcttggtaccccagcata-3′, which contains an artificial *BamH I* site; antisense, 5′- *gtcgac*ctgaaagggtgtggagtgct-3′, which contains an artificial *Sal I* site. The PCR-amplified fragments were then digested with *BamH I/Sal I* and inserted into the corresponding restriction sites of the *pGL3-β* plasmid described above to generate *pβ-3′E_κ_wt*. The PCR products were confirmed by their size, as determined by electrophoresis and by DNA sequencing. The PU motif mutant (designated as *pβ-3′E_κ_mt*) from *pβ-3′E_κ_wt* was generated by PCR based on an overlap extension technique [32]. The primers used for generating mutations were: forward, 5′-accctttgggcccctgaaaacagaacc-3′; reverse, 5′-ttttcaggggcccaaagggtcttctcc-3′. The PCR-amplified fragments carrying the desired mutations were then cloned into the *BamH I/Sal I* sites of the *pGL3-β* plasmid. The expected mutations and the integrity of the enhancer were confirmed by automated sequencing using an Applied Biosystems sequencer and software (Foster City, CA).

### RNA interference

HNE2-LMP1 cells were grown in 6-well plates and transfected with an ERK-specific small interfering RNA oligonucleotide (si-ERK; Cat no: #6560; 100 pmol; Cell Signaling) or scrambled oligonucleotides (si-scrambled; Cat no: #6568;100 pmol; Cell Signaling); an Ets-1-specific small interfering RNA oligonucleotide (si-Ets-1; Cat no: sc-29309; 150 pmol; Santa Cruz) or scrambled oligonucleotides (si-scrambled; Cat no: sc-37007; 150 pmol; Santa Cruz) using Lipofectamine™ 2000 (Invitrogen, USA) for 72 hr according to the manufacturer's instructions. To confirm ERK or Ets-1 knockdown, cells transfected with si-ERK, si-Ets-1, or scrambled oligonucleotide were harvested for protein extraction and immunoblotting.

### Luciferase reporter assays

The *pGL3-β, pβ-3′E_κ_wt* and *pβ-3′E_κ_mt* firefly luciferase reporter plasmids described above were used in conjunction with the control *pGL3-Basic* vector (Promega) and the internal control plasmid *pRL-SV40* (Promega). Cells were cultured in 24-well plates at a density of 1×10^5^ per well overnight and then transfected with the indicated plasmid using Lipofectamine™ 2000 (Invitrogen) following the manufacturer's instructions. Each transfection contained 800 ng/well of the firefly luciferase reporter plasmid and 80 ng/well of the internal control *pRL-SV40* plasmid. At 24 hr after transfection, cells were either left untreated or treated with 50 µM PD98059 or 0.1% DMSO for 12 hr. Cells were harvested at 36 hr after transfection and lysates were analysed for firefly and *renilla* luciferase activity according to the manufacturer's instructions using the Dual-Luciferase Reporter Assay Kit (Promega) with a GloMax 20/20 luminometer (Promega). The luciferase reporter plasmids were co-transfected with the *pRL-SV40* vector to correct for variations in transfection efficiency. The data are represented as the fold induction compared to the *pGL3-Basic* vector. At least three independent transfection experiments were performed in triplicate for each experimental construct.

### Reverse transcription and polymerase chain reaction

Subconfluent HNE2 and HNE2-LMP1 cells were treated or not treated with 50 µM PD98059 for 12 hr. Total RNA was isolated from the cells, including XG6, XG7 or Raji cell lines as controls, using the TRIzol reagent (Invitrogen) according to the instructions of the manufacturer. RNA was dissolved in 20 µl of DEPC-treated water and quantified at 260 nm. Total RNA (2 µg) was reverse transcribed with SuperScript™ IIRT (Invitrogen) at 42°C for 50 min, and the resulting cDNA was subjected to PCR. For *kappa light chain* RT-PCR, in order to determine the optimal PCR cycle number, a constant amount of input cDNA was used in PCR reactions, the cycle number was varied between 25 and 40 (25, 28, 32, 36, 38, 40) and the PCR product of 36 cycles showed a good detectable signal and was in the linear range. The cycling conditions for *human kappa light chain* (GenBank accession no. AJ010442) or for *actin*: 94°C for 5 min followed by 36 cycles of 94°C for 30 sec, 50°C for 30 sec, 72°C for 30 sec, and an extension for 10 min at 72°C. PCR thermocycling conditions for Ets family members, LMP1 and GAPDH are 95°C for 5 min followed by 32 cycles of 95°C for 30 sec, 55°C for 30 sec, 72°C for 40 sec, and an extension for 10 min at 72°C. For quantitative real-time RT-PCR (qRT-PCR), iTaq SYBR Green Supermix with Rox (Cat. no. 172-5850, Bio-Rad) was used with the following cycling conditions: 95°C for 10 min, then 40 cycles of 95°C for 15 sec followed by 60°C for 1 min in an ABI Prism 7500 Sequence Detection System (Applied Biosystems). The relative mRNA expression levels were calculated according to the comparative CT (ΔΔCT) method after normalizing to GAPDH expression. Primer sequences for amplification of Ig kappa light chain, Ets family members [33], LMP1 [34] and internal controls are listed in Table S1. PCR products were separated on 1.5–2% agarose gels and visualized with ethidium bromide.

### Protein extraction

Whole-cell lysates were essentially prepared according to a method previously described [2]. For the electrophoresis mobility shift assays (EMSAs), nuclear extracts were prepared using the NE-PER Nuclear and Cytoplasmic Extraction Kit (Cat. no. 78833, Pierce, USA) following the manufacturer's instructions. Protein concentration was determined using the BCA Assay Reagent (Cat. no. 23228, Pierce).

### Western blot analysis

Proteins (50 to 100 µg) were boiled in SDS sample buffer for 5 min, resolved on 10% SDS-polyacrylamide gels and transferred onto a nitrocellulose membrane (Millipore, USA). Nonspecific reactivity was blocked by incubating the membrane for 30 min in a Tris-buffered saline solution containing 0.1% Tween-20 and 10% nonfat dried milk. The membrane was incubated overnight at 4°C with various primary antibodies, followed by incubation at room temperature for 1 hr with a horseradish peroxidase-conjugated mouse or rabbit secondary antibody (Santa Cruz, USA), and then washed three times for 10 min each with Tris-buffered saline containing 0.1% Tween-20. The antibody-bound proteins were detected using an enhanced chemiluminescence detection kit (Cat. no. 34075, Pierce) followed by exposure to autoradiographic film. After probing for phosphorylated ERKs or phosphorylated Ets-1 to examine ERKs activation status or the level of phosphorylated Ets-1, membranes were stripped by incubating at 50°C for 30 min in stripping buffer (100 mM β-mercaptoethanol, 2% (wt/vol) sodium dodecyl sulfate and 62.5 mM Tris-HCl pH 6.8) and reprobed with anti-ERK or anti-Ets-1. The following antibodies were used for Western blotting: mouse anti-LMP1 monoclonal antibody (CS.1–4, DAKO, Denmark), rabbit anti-human kappa light chain antibody (A0191, DAKO), Ets-1 (sc-350), phosphorylated threonines (sc-5267), ERK (sc-93), phosphorylated ERK (sc-7383), and α-tubulin (sc-5286) (all from Santa Cruz).

### Immunoprecipitation

Whole-cell lysates (200 µg) were mixed with protein A-Sepharose beads (Sigma), incubated at 4°C for 2 hr, and centrifuged for 2 min at 2,000 rpm for preclearing. Then the supernatant fraction was incubated at 4°C overnight with 4 µg of an Ets-1 antibody and protein A-Sepharose beads, followed by centrifugation for 2 min at 12,000 rpm. The immunoprecipitates were collected and washed five times with PAPI buffer (50 mM Tris-HCl pH 7.5, containing 1% NP-40, 0.05% SDS, 0.5% sodium deoxycholate, 1 mM EDTA, 150 mM NaCl, and protease inhibitors). The precipitates were eluted from the protein A-Sepharose beads by boiling for 5 min and finally subjected to Western blot analysis using an antibody to detect threonine phosphorylation.

### Electrophoretic mobility shift assays

Electrophoretic mobility shift assays (EMSAs) were conducted using the LightShift™ Chemiluminescent EMSA Kit (Cat. no. 20148, Pierce) following the manufacturer's instructions. The protein concentration in nuclear extracts was determined using the BCA protein assay reagent (Cat. No. 23228, Pierce) and EMSAs were performed using aliquots containing equal amounts of protein. The reaction mixtures (20 µl) containing nuclear extracts (8 µg) were incubated with biotin-labeled double-stranded oligonucleotide probes (2 nM) in reaction buffer (Pierce) for 20 min at room temperature. Reactions were subjected to electrophoresis on 5% polyacrylamide gels in 0.5× Tris-borate-ethylene diamine tetra-acetic acid (TBE) buffer followed by electroblotting onto Biodyne™ B Nylon membranes (Cat. no. 77016, Pierce) and UV cross-linking. Biotinylated oligonucleotides were then detected by probing with streptavidin conjugated to HRP and visualized using an ECL kit and autoradiography. For competition analyses, a 100-fold excess of the corresponding unlabeled wild-type oligo or the mutant oligo was included in the binding reaction. For antibody supershift experiments, the reaction mixtures were preincubated with 2 µg of an Ets-1 (sc-350X, Santa Cruz) antibody at room temperature for 1 hr. The complementary oligonucleotides used as probes or competitors are as follows: wild-type human κPU oligonucleotides, 5′- gaagaccctttggggaactgaaaacaga-3′ and 5′-tctgttttcagttccccaaagggtcttc-3′, derived from the sequence of the PU site within the human 3′E_κ_. The mutated κPU oligonucleotides (designated as mutPU) used were 5′-gaagaccctttggg**ccc**ctgaaaacaga-3′ and 5′-tctgttttcag**ggg** cccaaagggtcttc-3′. The non-specific competing probes were 5′-ccagagggggatttccaagaggcca-3′ and 5′-tggcctcttggaaatccccctctgg-3′, which were derived from the sequence of the NF-κB site within the human iE_κ_. Binding sites are underlined and mutations are shown in bold type. The mutated oligo probes against the κPU binding site for EMSAs are identical to those of the mutated sequences in the reporter gene constructs.

### Chromatin immunoprecipitation (ChIP) assay

ChIP analysis was performed using a ChIP assay kit (Upstate Biotechnology, Lake Placid, NY) according to the manufacturer's recommendations. Briefly, a formaldehyde solution was added directly to HNE2-LMP1 cells at a final concentration of 1% and incubated at room temperature for 10 min. Then the cells were neutralized for 5 min with glycine at room temperature and washed twice with ice-cold 1× phosphate-buffered saline containing protease inhibitors. The cells were disrupted using SDS lysis buffer containing protease inhibitors. Chromatin in the lysate (350 µl) was sheared to an average length of ∼500 bp by sonication with a Branson Sonifier Cell Disruptor B15 (output control 4, duty cycle 40%), with 14 cycles of 20-sec pulses at 20-sec intervals. The suspension was precleared in a salmon sperm DNA/protein A/agarose-50% slurry for 1 hr at 4°C. After the chromatin was “precleared”, a small aliquot (10 µl) was saved as “input DNA” for PCR analysis later. The remaining aliquots (100 µl each) of sheared cross-linked chromatin were incubated overnight with 2 µg Ets-1 (sc-350X), rabbit IgG (sc-2027) (Santa Cruz), or no antibody at 4°C with mild shaking. The immune complexes were incubated for 2 hr at 4°C in a salmon sperm DNA/protein A/agarose-50% slurry with mild shaking, washed, and eluted. Cross-linking was reversed using 5 M NaCl. After proteinase K digestion, the DNA in the samples was extracted with phenol, precipitated with ethanol, and resuspended in 50 µl of ddH_2_O. The DNA solution (2 µl) was used for 36 cycles of PCR amplification. PCR products were analyzed by electrophoresis on a 2% agarose gel and visualized by ethidium bromide staining. The following primers were used in the ChIP assays: human 3′E_κ_ enhancer including the PU-binding region, 5′- ccagggaccaagatagcaac -3′ and 5′- ctgaaagggtgtggagtgct -3′ (158 bp).

### Statistical analysis

All statistical calculations were performed using the SPSS (v. 12.0) statistical software program. Differences between various groups were evaluated by the Student's *t* test. The difference was statistically significant when *p*<0.05.

## Results

### LMP1 enhances kappa light chain expression through the ERKs signaling pathway in human NPC cells

**Figure 1:**
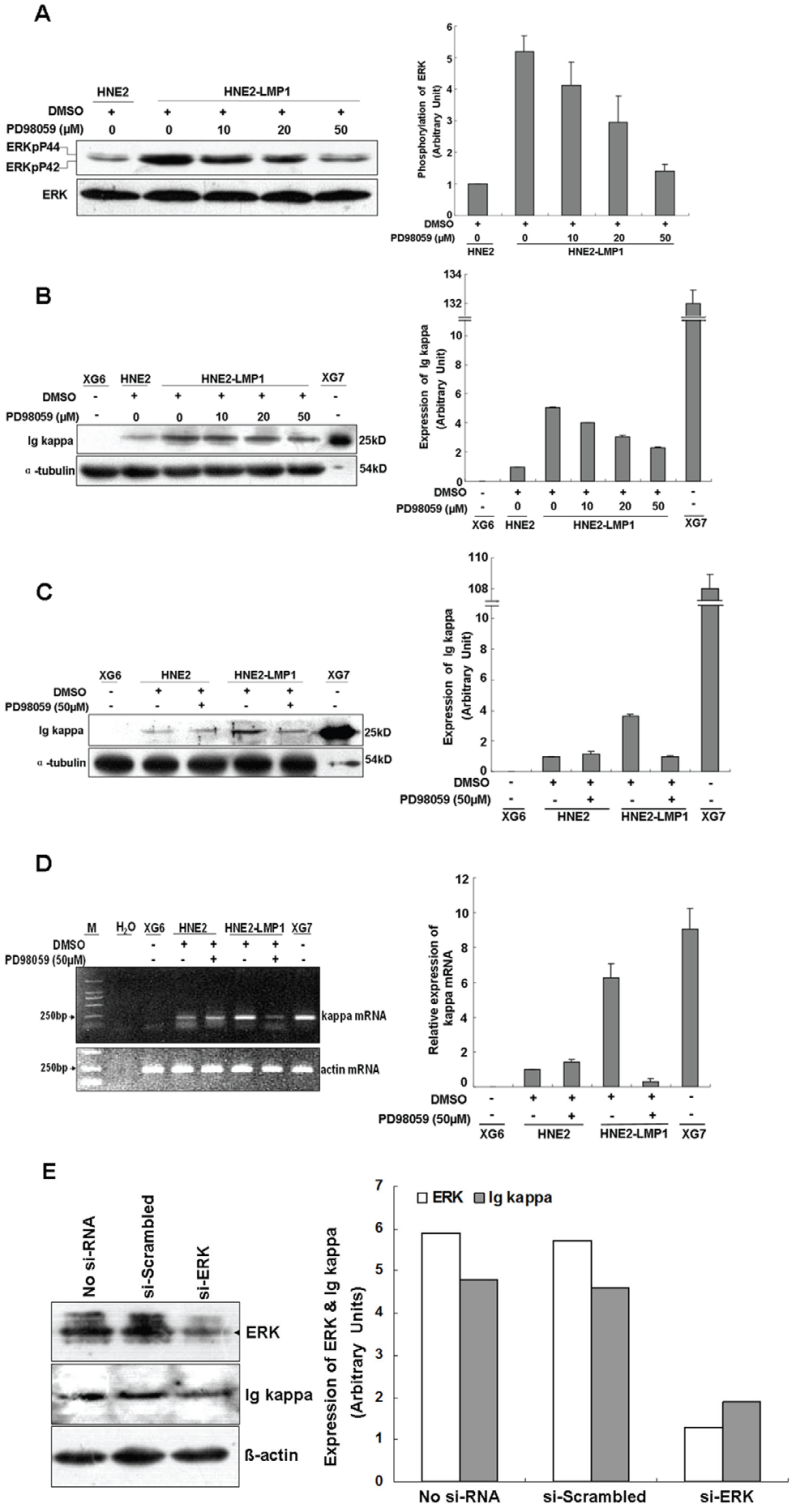
Inhibition of the ERKs signaling pathway blunts LMP1-increased kappa light chain expression at both the mRNA and protein levels. (A) HNE2-LMP1 cells were treated with the indicated concentrations of PD98059 or 0.1% DMSO for 2 hr. Whole cell lysates were prepared and total and phosphorylated ERKs levels were determined by Western blotting. (B) HNE2-LMP1 cells were treated with the indicated concentrations of PD98059 or 0.1% DMSO for 12 hr. Kappa light chain expression in NPC cells was assessed by Western blotting using a specific antibody. (C) HNE2 and HNE2-LMP1 cells were treated with 50 µM PD98059 or 0.1% DMSO for 12 hr and Western blotting was performed to detect kappa light chain expression. (D) HNE2 and HNE2-LMP1 cells were incubated with medium containing the indicated concentration of PD98059 or 0.1% DMSO for 12 hr. Total RNA was isolated from cells and subjected to RT-PCR, using specific primers designed to amplify *kappa light chain* and *actin* mRNAs. (E) HNE2-LMP1 cells were transfected with *si-ERK* or scrambled oligonucleotide. ERK and Ig kappa protein levels were detected by immunoblotting. The results shown are representative of three independent experiments. Phosphorylation or total expression level for each protein as well as mRNA was estimated by densitometry and are presented as a ratio to the respective loading control (right panels). XG7 and XG6 cells are shown as positive and negative controls, respectively, for kappa light chain.

LMP1 can activate the Ras/ERK/MAPK signaling pathway [23] and our previous study indicated that LMP1 upregulates kappa light chain expression in NPC cell lines [2]. To confirm whether ERKs pathway is involve in LMP1-augmented kappa light chain expression, PD98059 was used to manipulate ERKs activation and kappa chain upregulation induced by LMP1. The level of ERK phosphorylation was higher in HNE2-LMP1 cells than in HNE2 cells, which demonstrated that LMP1 indeed activates the ERK pathway in NPC cells (Figure 1A). Treatment of HNE2-LMP1 cells with PD98059 resulted in a dose-dependent suppression of LMP1-induced kappa light chain (Figure 1B), which corresponded with a dose-dependent attenuation of ERK phosphorylation induced by LMP1 (Figure 1A). PD98059 (50 µM) clearly showed an inhibitory effect on HNE2-LMP1 cells, but had no effect on HNE2 cells (Figure 1C). In order to determine if the inhibition of Ig kappa expression by PD98059 treatment occurs at the transcriptional level, HNE2 and HNE2-LMP1 cells were maintained under the same conditions and the levels of *kappa light chain* mRNA were examined by RT-PCR. Treatment with PD98059 (50 µM) induced a marked decrease in LMP1-induced *kappa* mRNA expression, but the *kappa* mRNA level in HNE2 remained essentially unchanged (Figure 1D), which agrees with the immunoblot results. To further confirm that the ERK pathway plays a role in LMP1-induced *kappa light chain* gene expression, we knocked down *ERK* expression by RNA interference. Whereas transfection of HNE2-LMP1 cells with a scrambled oligonucleotide did not affect LMP1-induced *kappa* gene expression, *si-ERK* blunted the effect of LMP1 (Figure 1E), indicating that the ERK pathway is involved in this event. Overall, these results confirm the idea that upregulation of kappa light chain by LMP1 occurs through the activation of the ERK/MAPK signaling pathway.

### The PU binding site is involved in LMP1-enhanced activity of 3′Eκ through the ERK pathway

**Figure 2:**
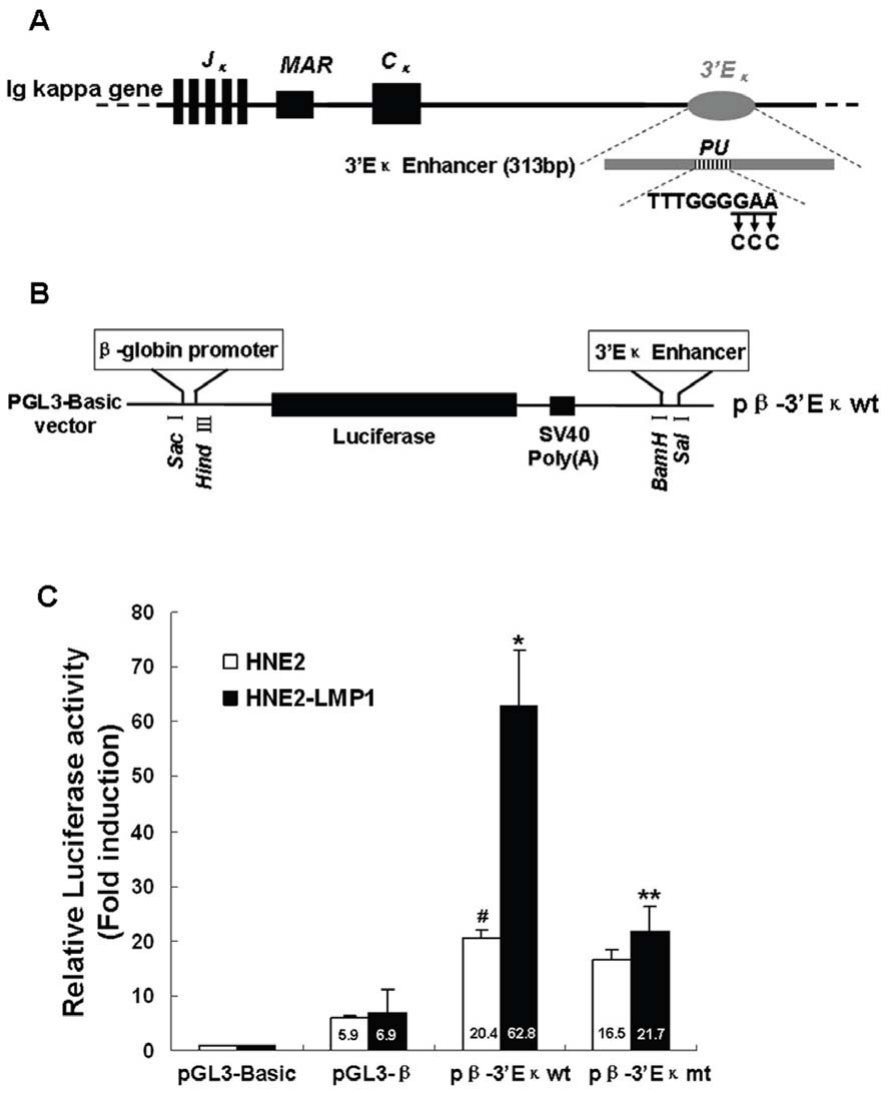
The PU binding site is involved in LMP1-induced human 3′E_κ_ enhancer activity in NPC cells. (A) Schematic diagram of the human 3′E_κ_ core fragment-containing DNA portion used in these experiments. The position of the PU binding site is shown. For simplicity, other protein-binding sites in the 3′E_κ_ are not shown. The expansion of the PU binding site gives its wild-type sequence and the nucleotides replaced by mutations are underlined. Arrows indicate the nucleotides introduced by mutations. (B) Insertion sites for the DNA fragment in the *pGL3-β* vector, which contains the human *β-globin* promoter and the *luciferase* reporter gene. (C) Comparison of 3′E_κ_ activity in human nasopharyngeal carcinoma cell lines. The constructs carrying the wild-type PU sequence (*pβ-3′E_κ_wt*), mutant PU sequence (*pβ-3′E_κ_mt*), *pGL3-β* or *pGL3-Basic* with the internal control plasmid *pRL-SV40* were transiently co-transfected into HNE2 and HNE2-LMP1 cells. Luciferase reporter assays were performed as described in “Materials and methods”. Values for firefly luciferase activity were normalized to those obtained for *Renilla* luciferase activity. Values obtained for cells transfected with *pβ-3′E_κ_wt, pβ-3′E_κ_mt* and *pGL3-β* were divided by the corresponding values obtained for cells transfected with *pGL3-Basic*. Data are shown as means ± S.D. of three independent experiments performed in triplicate. Statistical significance: #*p*<0.05 *vs. pGL3-β*-transfected HNE2 cells; *p<0.01 *vs. pGL3-β*-transfected HNE2-LMP1 cells; **p<0.05 *vs. pβ-3′E_κ_w*t-transfected HNE2-LMP1 cells.

**Figure 3:**
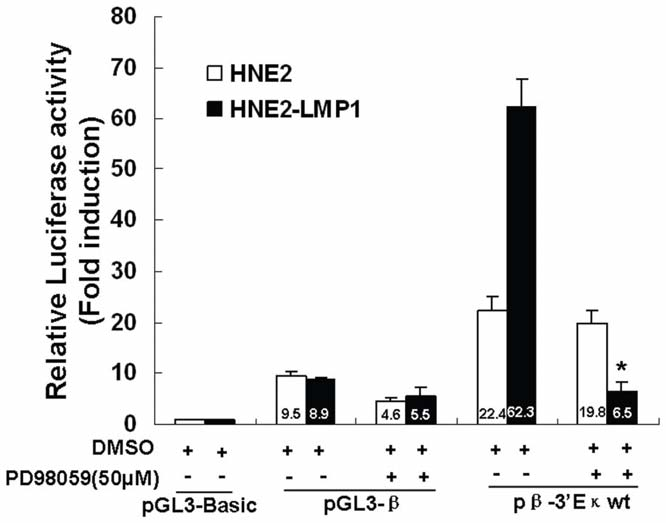
The MEK inhibitor, PD98059, abolishes the LMP1-increased 3′E_κ_ enhancer activity. HNE2 and HNE2-LMP1 cells were co-transfected with *pβ-3′E_κ_wt, pGL3-β,* or *pGL3-Basic* and the internal control *pRL-SV40* plasmids. Cells were incubated for 24 hr and then treated with PD98059 (50 µM) or DMSO (0.1%) for an additional 12 hr after which activity of firefly and *Renilla* luciferase was monitored as described in “Materials and Methods”. Values for firefly luciferase activity were normalized to those obtained for *Renilla* luciferase activity. Values obtained for *pβ-3′E_κ_wt-* or *pGL3-β*-transfected cells were divided by the corresponding values obtained for *pGL3-Basic-*transfected cells. Data are shown as means ± S.D. of three independent experiments performed in triplicate. Statistical significance: *p<0.05.

The activation of the 3′E_κ_ is required for *immunoglobulin kappa* gene expression. In order to investigate whether 3′E_κ_ could be functionally activated in NPC cells, we linked a 313 bp human 3′E_κ_ enhancer fragment, which contains the enhancer core and 90 bp upstream of the enhancer core sequences that is necessary for maximal enhancer activity when the enhancer core is not directly adjacent to the promoter [31], to the human *β-globin* promoter to drive expression of the firefly luciferase gene and analyzed this reporter construct by transient transfection of NPC cell lines (Figure 2A, B). The human *β-globin* promoter was chosen because it has previously been used for several studies of immunoglobulin enhancers [35], [36], [37], and also because we found it to be minimally affected by LMP1 in our experiments (Figure 2C and Figure 3). The constructs were introduced into HNE2 and HNE2-LMP1 cells to test the activity of 3′E_κ_. Transfection of *pβ-3′E_κ_wt* generated higher luciferase activity than transfection of the *pGL3-β* construct (no enhancer), regardless of whether LMP1-negative (*p*<0.05) or LMP1-positive (*p*<0.01) NPC cells were examined. These results indicated that 3′E_κ_ is active in NPC cells that expressed the Ig kappa light chain. Moreover, the activity of 3′E_κ_ in HNE2-LMP1 cells was approximately 3-fold higher than that in HNE2 cells (*p*<0.05) (Figure 2C), which agreed with the kappa chain expression patterns of these two cell lines [2]. Notably, the luciferase activity of *pGL3-β* in both HNE2 and HNE2-LMP1 cells was essentially equivalent, which suggested that the difference in the 3′E_κ_ activity between HNE2 and HNE2-LMP1 cells was due to the enhancer itself rather than the promoter sequence. These results indicate that 3′E_κ_ is active in Ig kappa-expressing NPC cells and LMP1 elevates the activity of 3′E_κ_, which might account for the upregulation of the kappa light chain protein by LMP1 in NPC cells.

PU motif (TTTGGGGAA) is one of positive regulatory elements that modulate kappa light chain expression in B cells [10]. To confirm the role of this element in regulating kappa light chain expression in NPC cells, site-directed mutagenesis by overlap extension PCR was used to introduce mutations into the consensus PU motif for the family of transcription factor Ets-related proteins within 3′E_κ_. The *pβ-3′E_κ_mt* construct was created and used to test the activity of 3′E_κ_. Mutation of the PU motif significantly (*p*<0.05) decreased LMP1-increased 3′E_κ_ activity (Figure 2C). In contrast, the 3′E_κ_ activity in HNE2 cells was slightly decreased by this genetic manipulation. This result and the result showing that mutation of the PU motif could not completely abolish 3′E_κ_ activity in HNE2-LMP1 cells, as well as previous reports indicating that several additional functional motifs reside within 3′E_κ_ [10], [11], suggest that a variety of nuclear factors can bind to 3′E_κ_ resulting in highly complex regulatory pathways. Together, the results indicated that the PU binding site contributes to the 3′E_κ_-mediated *kappa light chain* gene transcriptional activation.

To further confirm that the LMP1-induced transcriptional enhancement effect on 3′E_κ_ is ERK-mediated, we used the ERK upstream kinase inhibitor, PD98059, to block ERK signaling and test 3′E_κ_ activity. LMP1-induced activity of 3′E_κ_ was dramatically inhibited (p<0.05) by PD98059 (50 µM, Figure 3). The inhibitory effect of PD98059 on LMP1-induced 3′E_κ_ activity was more robust than the inhibition resulting from the mutation in the PU binding site (Figure 2C and Figure 3). One possible explanation for this observation is that other transcription factors that regulate 3′E_κ_ activity are likely ERK-targeted substrates, which can be simultaneously inhibited by PD98059. This compound also decreased 3′E_κ_ activity in HNE2 cells to a certain extent, but the decrease was not statistically significant (*p*>0.05), which is consistent with the immunoblot (Figure 1C) and RT-PCR (Figure 1D) results shown earlier. We speculated that PD98059 can not reduce the basal activity of ERKs as did NF-κB inhibitor Bay11-7082, which was reported that did not reduce the basal activity of an NF-κB [38]. This could explain why PD98059 had no obvious effect on HNE2 cells. The transcriptional activities of the human *β-globin* promoter were slightly decreased by PD98059 in both LMP1-negative and LMP1-positive NPC cells. Based on these results, we concluded that the PU binding site contributes to LMP1-increased 3′E_κ_ activity as well as *kappa light chain* gene expression and these events are mediated by ERK signaling.

### *Ets* family gene expression in human NPC cell lines

**Figure 4:**
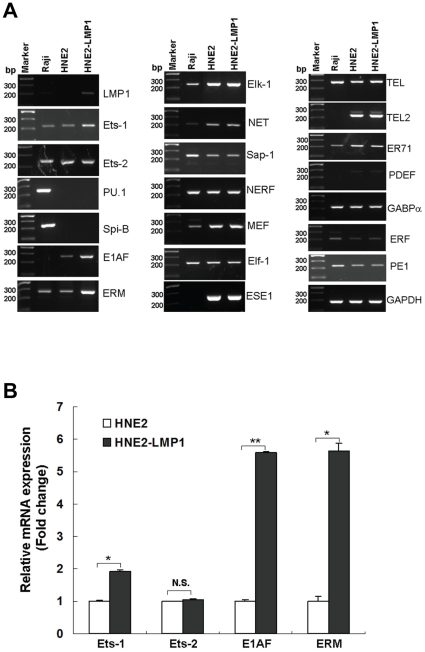
ETS family gene expression in human NPC cells. (A)Total RNA was isolated from HNE2, HNE2-LMP1 and Raji cells and the expression of ETS family members at the mRNA level was analyzed by RT-PCR. The B-cell lineage Raji cell line was used as a control. *GAPDH* was used as an internal control. (B) mRNA expression levels for *Ets-1*, *Ets-2, E1AF* and *ERM* in HNE2 and HNE2-LMP1 cells were analyzed by quantitative real-time RT-PCR. The level of each mRNA from HNE2 was normalized to a value of 1. Fold-change in mRNA levels is shown. Values are presented as the mean ± S.D of two independent experiments performed in triplicate. Statistical significance: *p<0.01, **p<0.001.

To characterize which Ets family member bound to PU motif in human NPC cells, we first analyze *Ets* family gene expression in these cell lines. There are 27 *Ets* genes in the human genome. Genome-wide analyses reveal properties of redundant within the *Ets* gene family members [19]. We tested mRNA level of *Ets* gene family members, including those that are classified as ubiquitous or cell-type-specific expressions [33]. The RT-PCR results showed that two hematopoietic tissue-specific Ets transcription factors *PU.1* and *Spi-B*, which are important for B cell maturation, were highly expressed in Raji cells but were not detectable in two NPC cell lines. Consistent with previous report [33], mRNA levels of *E1AF*, *ESE1*, *TEL2* and *PDEF* in Raji cells were too low to be detected. In addition, RT-PCR results showed that the mRNA levels of *Ets-1, E1AF* and *ERM* were higher in HNE2-LMP1 cells than those in HNE2 cells. Other Ets family members assessed, such as *Elf-1*, *Elk-1*, *Sap-1*, showed no significant difference at mRNA levels between HNE2 and HNE2-LMP1 cells (Figure 4A). We also performed quantitative real-time RT-PCR to compare mRNA levels of *Ets-1*, *Ets-2*, *E1AF* and *ERM* genes between HNE2 and HNE2-LMP1 cells. As shown in Figure 4B, higher mRNA levels of *Ets-1*, *E1AF* and *ERM* in HNE2-LMP1 cells, about a 2-fold increase for *Ets-1* as well as 5-fold increases for *E1AF* and *ERM,* compared with HNE2 cells. No significant difference of *Ets-2* mRNA level between HNE2 and HNE2-LMP1 cells. The *Ets-2* mRNA expression pattern in HNE2 and HNE2-LMP1 cells was in accordance with a previous report that the level of Ets-2 protein was unaffected by LMP1 [39]. The result indicated that LMP1 induced *Ets-1*, *E1AF* and *ERM* expression at mRNA level.

### Upregulation of kappa light chain expression by LMP1 occurs through ERK-mediated Ets-1 phosphorylation and activation

**Figure 5:**
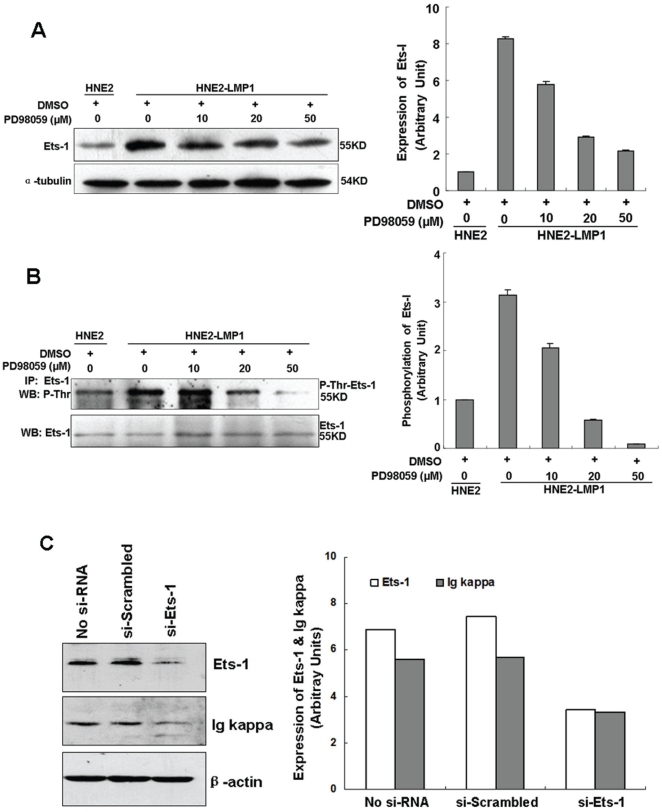
Inhibition of ERKs signaling reduces LMP1-induced Ets-1 expression and phosphorylation. (A) Inhibition of LMP1-upregulated Ets-1 protein expression by the MEK inhibitor, PD98059. HNE2-LMP1 cells were treated with the indicated concentrations of PD98059 or 0.1% DMSO for 12 hr. Ets-1 expression in NPC cells was determined by Western blot. A representive image of three independent experiments with similar results and the Ets-1 expression level quantified by densitometry are shown. α-Tubulin was used to verify equal protein loading. (B) Inhibition of threonine phosphorylation of Ets-1 by the MEK inhibitor, PD98059. NPC cells were treated with the indicated concentrations of PD98059 for 12 hr and whole cell extracts were immunoprecipitated with an anti-Ets-1 antibody. Similar protein levels of Ets-1 were loaded and resolved by SDS-PAGE, and the levels of Ets-1 threonine phosphorylation were determined by Western blot analysis using a pan threonine phosphorylation antibody. The membrane was then stripped and reprobed with anti-Ets-1. The threonine phosphorylation level of Ets-1 was quantified by densitometry. (C) HNE2-LMP1 cells were transfected with *si-Ets-1* or scrambled oligonucleotide. Ets-1 and Ig kappa protein levels were detected by immunoblotting. The results shown are representative of three independent experiments. Phosphorylation or expression level for each protein was estimated by densitometry and was presented as a ratio to the respective loading control (right panels).

Ets-1 is a nuclear target of the Ras signaling pathway and contains an ERK MAP kinase docking site [26], [40]. The phosphorylation of Ets-1 is a necessary molecular event for Ras-mediated activation of this transcription factor [26]. To investigate whether the transcription factor Ets-1 plays an important role in LMP1 upregulation of kappa light chain expression through ERK pathway, Ets-1 protein expression, phosphorylation and activation were analyzed. A higher level of Ets-1 protein expression and phosphorylation was observed in HNE2-LMP1 cells (Figure 5A, B), indicating that LMP1 could promote both increased expression and phosphorylation levels of Ets-1. To examine whether LMP1-increased expression and activation of Ets-1 were also mediated by the ERK signaling pathway, the contribution of ERK was evaluated through the inactivation of ERK activity. The result showed that PD98059 reduced LMP1-induced upregulation of Ets-1 protein expression in a dose-dependent manner (Figure 5A). We then evaluated the phosphorylation status of Ets-1 under the same treatments. Ets-1 proteins were immunoprecipitated and similar levels of Ets-1 were loaded to avoid the possibility that the differences in the protein level of Ets-1 interfere in Ets-1 phosphorylation levels [41]. Western blotting analysis of Ets-1 immunoprecipitates with a pan threonine-phospho-specific antibody indicated that treatment with PD98059 resulted in a concentration-dependent inhibition of LMP1-induced Ets-1 threonine phosphorylation (Figure 5B). This result corresponded with a dose-dependent attenuation of LMP1-induced ERK phosphorylation (Figure 1A) and LMP1-induced kappa light chain expression (Figure 1B). These data indicated that ERK might modulate activation of Ets-1 by modifying Ets-1 turnover as well as through its phosphorylation. To further determine whether Ets-1 is involved in LMP1-induced Ig kappa expression, we evaluated the effect of Ets-1 siRNA on Ig kappa expression in HNE2-LMP1 cells. The siRNA-mediated knockdown of *Ets-1* gene expression was verified by immunoblot analysis (Figure 5C). Simultaneously, we found that the knockdown of endogenous *Ets-1* is accompanied by a decrease of Ig kappa light chain expression level as compared with control siRNA (Figure 5C), pointing to a role of Ets-1 in kappa light chain regulation. Collectively, these findings suggest a probable signaling pathway by which LMP1 upregulates Ig kappa light chain expression by activating ERK to mediate Ets-1 expression and phosphorylation.

### LMP1 promotes binding of the Ets-1 transcription factor to the κPU motif *in vitro*

**Figure 6:**
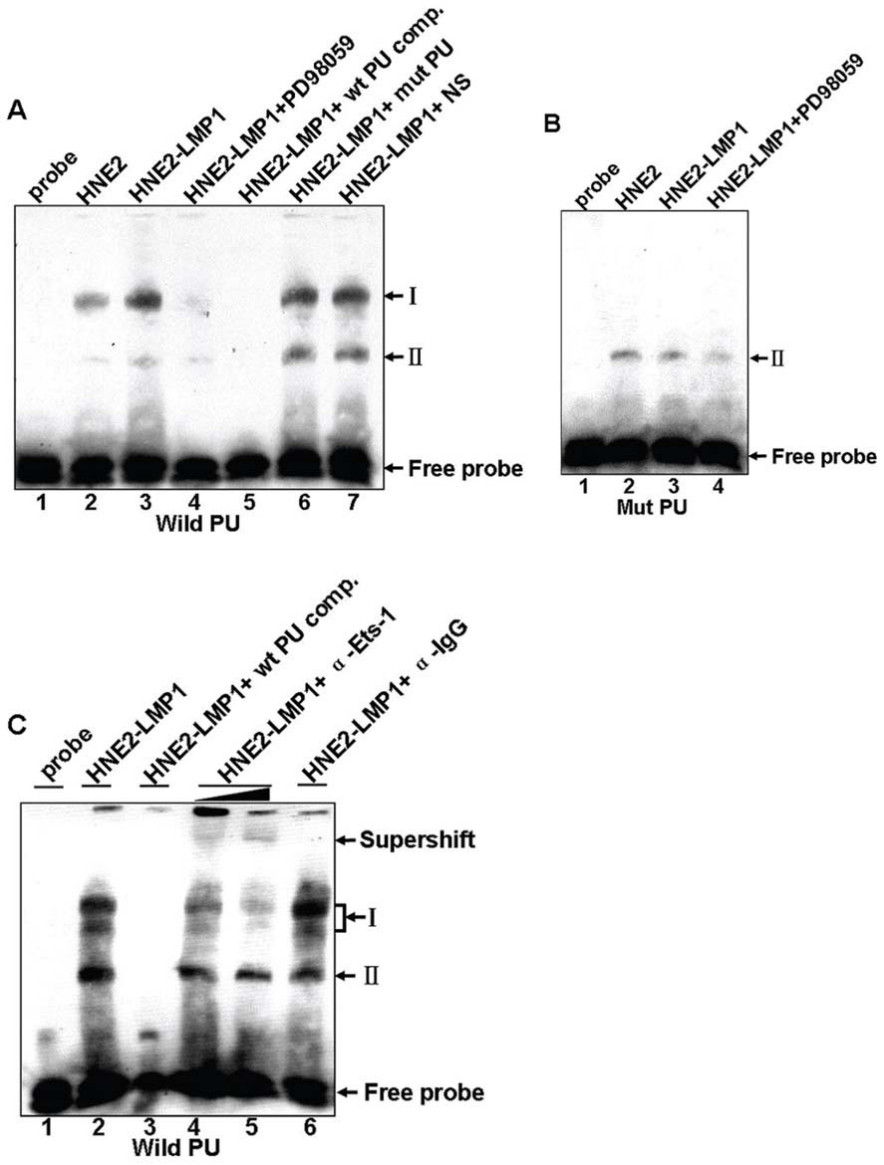
LMP1 enhances the binding ability of the Ets-1 transcription factor to human 3′E_κ_ enhancer through the ERKs signaling pathway *in vitro*. (A) A biotin-labeled wild-type κPU oligonucleotide probe was incubated with nuclear extracts of HNE2, HNE2-LMP1 and PD98059-treated HNE2-LMP1 cells (50 µM for 12 hr) in the presence of a 100-fold excess of unlabeled wild-type κPU (lane 5), a 100-fold excess of unlabeled mutant κPU oligonucleotide (designated mut PU, lane 6), or a non-specific competitor (NS, κNF-κB probe, lane 7). Protein-DNA binding activities were then examined by EMSA. (B) A biotin-labeled mutant κPU oligonucleotide probe was incubated with nuclear extracts of various NPC cell lines, and then protein DNA-binding activities were examined by EMSA. (C) In the binding assays, an Ets-1 antibody was preincubated with nuclear extracts from HNE2-LMP1 cells before the addition of the biotin-labeled wild-type κPU oligonucleotide probe. Super-EMSA was then performed. Protein-DNA complexes are indicated as I and II. See Materials and methods for the sequence details of the probes used.

We next examined whether the DNA fragment encompassing the PU motif of the human *kappa light chain* gene exhibits transcription factor binding activity and whether LMP1 can increase the binding through ERK signaling. Biotin-labeled double-stranded oligonucleotide probes containing the PU motif were used for EMSA analysis of binding in nuclear extracts from vehicle- or PD98059-treated HNE2 and HNE2-LMP1 cells. Results indicated that two different DNA/protein complexes (I and II) could be observed (Figure 6A). A 100-fold excess of unlabeled wild-type κPU probe (lane 5) successfully competed with the formation of the complex. However, a 100-fold excess of unlabeled mutant κPU probe (lane 6) or a non-specific NF-κB probe (lane 7) had no effect on the binding, thus demonstrating that both complexes were formed specifically. The nuclear lysates isolated from these cells did not induce the formation of complex I when a biotin-labeled mutant-type PU oligonucleotide was introduced, whereas complex II remained (Figure 6B), thus revealing that the Ets-related protein formed the slower migrating complex I. Moreover, pre-incubation of nuclear extract with an Ets-1 antibody resulted in the formation of supershifts accompanied by a dose-dependent attenuation of the intensity of complex I but no change in complex II (Figure 6C, lanes 4 and 5). The addition of an unrelated antibody did not alter the binding (Figure 6C, lane 6). These results indicated that the κPU site is capable of binding the Ets-1 transcription factor in nuclear extracts of NPC cells. We found a much higher level of complex I in HNE2-LMP1 cells compared to the level observed in HNE2 cells (Figure 6A, lane 2 *vs.* 3), which indicated that LMP1 could substantially increase Ets-1 transcription factor binding to the PU motif of the human 3′E_κ_ enhancer. The MEK inhibitor, PD98059 (50 µM), completely inhibited LMP1-induced Ets-1-DNA binding activity (Figure 6A, lane 4), but did not obviously alter the binding of PU oligonucleotide with HNE2 nuclear extract (Figure S1). Because the PU probe used was derived from the PU sequence in human 3′E_κ_, we speculated that LMP1 induced the Ets-1 transcription factor to target to 3′E_κ_ through the ERKs signaling pathway.

### LMP1 promotes Ets-1 binding to PU motif of human *kappa* gene in vivo

**Figure 7:**
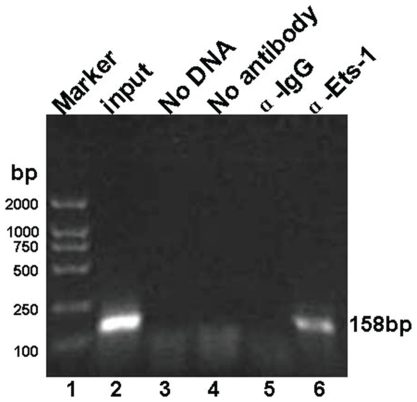
The transcription factor Ets-1 interacts with the human Ig *kappa* 3′ enhancer in cells. ChIP analysis of the PU binding site within the 3′ enhancer in HNE2-LMP1 cells. The cross-linked chromatin was precipitated with an Ets-1 antibody. The positive control is represented by the input fraction. Negative controls included a no chromatin sample, no antibody sample, and a nonspecific antibody (αIgG) sample. Precipitated DNA was analyzed by PCR using primers that amplified a 158-bp region, which included the PU binding site.

To better understand the role of the Ets-1 transcription factor in regulating human 3′E_κ_ and kappa light chain expression in cells, we analyzed the fragment that spans the PU binding region within 3′E_κ_ using a chromatin immunoprecipitation assay (ChIP). HNE2-LMP1 cells were treated with 1% formaldehyde to cross-link the proteins to chromatin and the cross-linked chromatin was then sheared by sonication to fragments of ∼500 bp in length. The sheared cross-linked chromatin was subsequently subjected to immunoprecipitation reactions using antibodies specific for detecting Ets-1. An IgG antibody was used as a nonspecific control. The precipitated chromatin DNA was then purified and amplified by PCR using primers specific for the PU binding site of the *Ig kappa* gene. The primers for the human 3′E_κ_ region containing the PU binding site produced 158-bp amplicons that could be observed with the positive control (input chromatin) and when the chromatin was precipitated with antibodies specific for Ets-1 (Figure 7). No amplification was observed with three negative controls (no chromatin, no antibody, and IgG). Therefore, the ChIP results indicated that the Ets-1 transcription factor can exert its regulatory function through direct binding to the human 3′E_κ_ enhancer and finally upregulating the kappa light chain expression in NPC cells. Overall, the results suggested that the interaction of Ets-1 with the PU binding site of the human *Ig kappa* gene might be a key event in the upregulation of kappa light chain expression by LMP1 in NPC cells.

## Discussion

The expression of Igs is widespread in epithelial cancers from many organs and basically includes all kinds of isotypes. Among the heavy chain class, the α chain for IgA and the γ chain for IgG were the most prevalent, whereas the κ, but not the λ, light chain was confirmed to be present. In our previous and present studies, we confirmed the aberrant expression of the Ig kappa light chain in NPC cells. Moreover, several studies indicated that tumor-derived Igs have certain biological functions. Qiu [4] found that blockade of tumor-derived IgG, expressing kappa light chain, using either antisense oligodeoxynucleotide or anti-human IgG, induced cancer cell apoptosis and inhibited cancer growth. This therefore confirmed that IgG secreted by epithelial cancers had some unidentified capacity to promote the growth and survival of tumor cells. We also found that obstruction of cancer-derived Ig alpha suppressed the growth and viability of cancer cells. Furthermore, we demonstrated that cancer-derived Ig alpha promotes the malignant proliferation ability of cancer cells and increases the number of cells in S phase from the early mitosis of synchronized cancer cells [42]. These findings support an important role for cancer-derived Ig as a growth factor of cancer cells. In addition, we found that the expression of *kappa constant region* mRNA was markedly increased in uterine cervical epithelia with dysplasia and carcinoma, as compared with cervicitis, thus suggesting a close association of kappa light chain expression with cell malignancy and with increasing tumor grades [1]. The fact that different research groups reported that nonlymphoid cells express Igs and the potential biological functions of the tumor-derived Igs suggests that this phenomenon is not a chance event. However, the mechanisms underlying the expression of Igs in nonlymphoid cells are still unknown. In the present study, we used Ig kappa-expressing NPC cell lines as models and focused mainly on exploring the possible mechanisms by which nonlymphoid cells express Ig kappa. We demonstrated that LMP1 promotes active Ets-1 binding to 3′E_κ_ that is mediated through the ERK signaling pathway, thus contributing to kappa light chain upregulation in NPC cells. Such mechanisms would explain, at least in part, the role of immunoglobulins produced in LMP1-positive human epithelial cancer cells.

The activation of *kappa* enhancers, whose function is mediated by proteins binding to the enhancers, is required for *Ig kappa* gene expression [43], [44], [45]. We found that 3′E_κ_ is active in both LMP1-negative and LMP1-positive NPC cells. In LMP1-negative HNE2 cells, the 3′E_κ_ activity is relatively small, which corresponds with the low kappa expression level. LMP1 can further stimulate 3′E_κ_ activity and contributes to the upregulation of Ig kappa in NPC cells. Our results indicated that mutation of the PU biding site did not completely abolish LMP1-induced 3′E_κ_ activity and only slightly attenuated 3′E_κ_ activity in HNE2 cells (Figure 2C). In addition to the PU motif in human 3′E_κ_ that modulates enhancer activity, the 3′E_κ_ enhancer region also comprises several putative sequences, including the κB motif, the interferon response element, CRE, PIP and E-box motifs for which functions have not yet been demonstrated. These sequences could potentially regulate 3′E_κ_ activity [10], [11]. Therefore, in addition to the Ets family proteins, other transcription factors binding to the 3′E_κ_ enhancer region mediated through various signaling pathways to regulate kappa expression in NPC cells cannot be excluded at this time.

By gel shift analysis, we found that the transcription factor Ets-1 bound to the κPU motif in NPC cells. The expression patterns of Ets family proteins are variable. Some are expressed ubiquitously, whereas others are expressed in a tissue-specific manner [15]. For example, PU.1 is exclusively expressed in hematopoietic cells [18]. In pre-B and B cells, PU.1 has been implicated in regulating the activities of enhancers and promoters in many B cell specific genes, including the *immunoglobulin kappa* gene. Intriguingly, the transcription factor Spi-C (Prf), which is closely related to PU.1 and Spi-B, can occupy PU binding sites [46]. An *in vitro* binding study demonstrated that two Ets family members, Ets-1 and Elf-l, could bind to the PU motif within 3′E_κ_ [47]. By investigating the endogenous ETS proteins Ets-1, Elf-1, and GABPα in a human T-cell line, Graves [19] discovered that these divergent family members frequently occupied the same genomic regions. The finding suggested that other Ets-related proteins were capable of binding to the PU site and contributed to *kappa* gene expression. In effect, our EMSA results demonstrated that, in NPC cells, the transcription factor Ets-1, but not PU.1, bound to the PU consensus sequences within the 3′E_κ_. In the absence of PU.1 in NPC cells, transcription factor Ets-1 might be more likely to compensate for the loss of PU.1 and occupy the PU binding site as well as play an important role in upregulating *kappa* gene expression. LMP1 also increased the mRNA levels of *E1AF* and *ERM*, members of the PEA3 subfamily of Ets transcription factors (Figure 4A, B). Whether or not they play a role in upregulating *kappa* gene expression in NPC cells remains to be elucidated. Additionally, our studies did not evaluate the phosphorylation status of other Ets family proteins in HNE2 and HNE2-LMP1 cell lines, and the possibility cannot be excluded that the phosphorylation levels but not expression levels of these proteins induced by LMP1, is involved in upregulating *kappa* gene expression in NPC cells.

We found that incubating the labeled κPU probe with nuclear extracts of NPC cells produced two protein-DNA complexes (I and II) (Figure 6). The κPU probes used in our experiments contained a wild-type IRF motif (GAAAAC) [48], which is located 2 bp downstream from the κPU motif. These two motifs form an Ets-IRF composite element (EICE), which has been identified in not only the *Igκ*, but also the *Igλ, IL-1β enhancers*, *CD20, gp91^phox^*, and *toll-like receptor 4 (TLR4)* promoters, and play an important role in gene regulation [49], [50]. The IRF transcription factor family comprises at least ten members. The expression of IRF-1, IRF-2, IRF-3, IRF-7 and IRF-9 is ubiquitous but IRF-4 and IRF-8 are thought to be expressed exclusively in cells of macrophage and lymphocyte lineages [50]. PU.1 was reported to interact with either IRF-4 or IRF-8 at the EICE element, resulting in transcriptional activation. IRF-4 can form ternary complexes on EICE with PU.1 or with another Ets-related factor, Spi-B [49]. IRF-8 can also form transcriptional complexes on EICE in a PU.1- or Spi-B-dependent manner [51], [52], [53]. These observations suggested that the complexes formed by Ets and IRF family members, which bind to the EICE element, might have great diversity. Our EMSA experiments indicate that Ets-1 can bind to the EICE element and complex I was, in effect, composed of two EMSA bands (Figure 6C) and the addition of anti-Ets-1 attenuated these two bands (Figure 6C, lanes 4 and 5). Therefore, we propose that Ets-1/DNA and Ets-1/IRFs/DNA complexes might exist. When assayed with a labeled mutant κPU probe, the whole complex I disappeared completely, which might be due to the failure of Ets-1 binding to the mutant κPU probe to form the Ets-1/DNA and Ets-1/IRFs/DNA complexes. Notably, in the mutant κPU probe, the wild-type IRF motif remained intact, which was in accordance with the observation that complex II was unaffected. We therefore speculated that the complex II might be an IRFs/DNA complex. In B cells, the PU.1/IRF-4 transcription factor complex binding to the *kappa* EICE element plays an important role in *kappa* gene regulation. However, the lack of haematopoietic cell lineage-specific transcription factors, PU.1 and IRF-4, in NPC cell lines, combined with our EMSA results leads us to speculate that the transcription factor Ets-1 might cooperate with other IRFs to bind the EICE element of the 3′E_κ_ to modulate *kappa* gene expression in a manner different from B cell lines. However, what kind of IRFs involved in forming the Ets-1/IRFs/DNA complexes and the mechanism by which Ets-1 interacts with IRFs as well as how these interactions relate to the control of 3′E_κ_ function in NPC cells will require further studies.

Expression of the kappa light chain in cancer cells is complicated. Not only the NPC cell lines, but many human epithelial cancer cell lines, including MCF-7 (breast carcinoma), HeLa (cervical carcinoma), MGC (gastric carcinoma), SW480 (colon carcinoma), and A549 (lung cancer) cells, express kappa light chain [1], [4]. Several infectious agents, particularly viruses, can encode oncoproteins, such as EBV-encoded LMP1, HBV-encoded X protein, HCV-encoded Core protein, HPV-encoded E6 and E7 proteins, have been identified as causes of cancer [54]. In NPC, LMP1 is considered to be a major oncogenic protein encoded by EBV and can aberrantly activate many signaling pathways. Similar to LMP1, other virus-encoded oncoproteins, including HBX, E6, and E7, might also be able to induce *immunoglobulin* gene expression in nonlymphoid cell lines by abnormally activated corresponding transcription factors and signaling pathways. The present study, using the NPC cell lines as models, provides some hints of possible mechanisms by which human cancer cells of epithelial origin produce immunoglobulins and lay the foundation for further studies.

## Supporting Information

Figure S1 **Effect of ERK inhibitor PD98059 on the binding ability of the Ets-1 transcription factor to human 3′Eκ enhancer in HNE2 cells** ***in vitro*** **.** A biotin-labeled wild-type κPU oligonucleotide probe was incubated with nuclear extracts of vehicle- or PD98059-treated HNE2 cells (50 µM for 12 hr) in the presence of a 100-fold excess of unlabeled wild-type κPU (lane 4), a 100-fold excess of unlabeled mutant κPU oligonucleotide (mutPU, lane 5), or a non-specific competitor (NS, κNF-κB probe, lane 6). Protein-DNA binding activities were then examined by EMSA. See Materials and methods for the sequence details of the probes used.
(DOC)

Table S1 **Primers used for amplification of human Ig kappa light chain, Ets family members and LMP1.**
(DOC)
