# Supplementary figures and images for: EBV-Encoded LMP1 Upregulates Igκ 3′Enhancer Activity and Igκ Expression in Nasopharyngeal Cancer Cells by Activating the Ets-1 through ERKs Signaling
Source: PLoS One. 2012 Mar 1;7(3):e32624. doi: 10.1371/journal.pone.0032624 (PMC3291551; doi:10.1371/journal.pone.0032624)

**Figure S1**


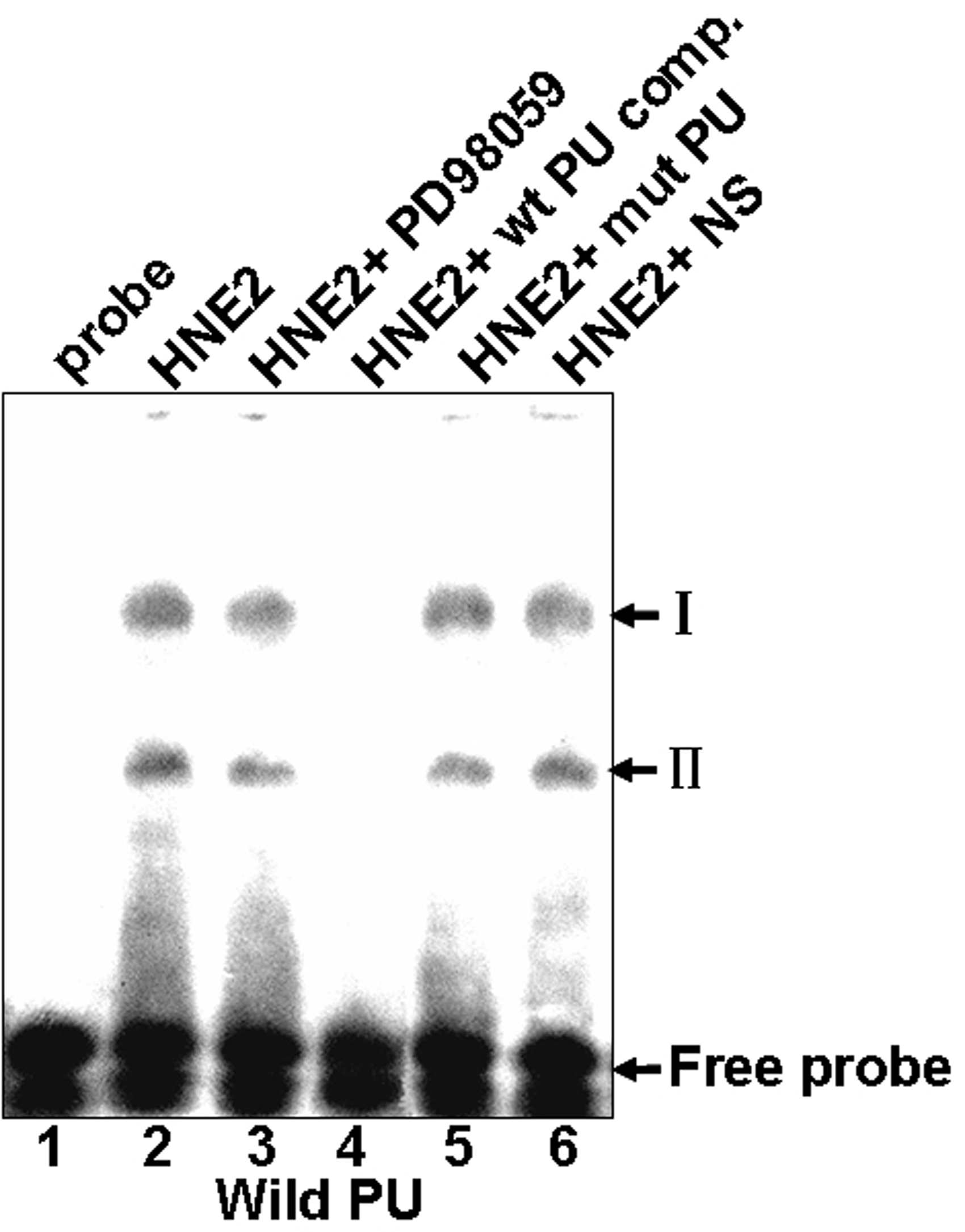

Supplement: Figure S1 — Effect of ERK inhibitor PD98059 on the binding ability of the Ets-1 transcription factor to human 3′Eκ enhancer in HNE2 cells in vitro . A biotin-labeled wild-type κPU oligonucleotide probe was incubated with nuclear extracts of vehicle- or PD98059-treated HNE2 cells (50 µM for 12 hr) in the presence of a 100-fold excess of unlabeled wild-type κPU (lane 4), a 100-fold excess of unlabeled mutant κPU oligonucleotide (mutPU, lane 5), or a non-specific competitor (NS, κNF-κB probe, lane 6). Protein-DNA binding activities were then examined by EMSA. See Materials and methods for the sequence details of the probes used. (DOC) [file pone.0032624.s001.doc]
