# Supplementary material for: EBV-Encoded LMP1 Upregulates Igκ 3′Enhancer Activity and Igκ Expression in Nasopharyngeal Cancer Cells by Activating the Ets-1 through ERKs Signaling
Source: PLoS One. 2012 Mar 1;7(3):e32624. doi: 10.1371/journal.pone.0032624 (PMC3291551; doi:10.1371/journal.pone.0032624)
Supplement: Table S1 — Primers used for amplification of human Ig kappa light chain, Ets family members and LMP1. (DOC) [file pone.0032624.s002.doc]

**Table S1. Primers used for amplification of human Ig kappa light chain, Ets family members and LMP1**

| **Gene** | **Sense sequence(5'to3')** | **Antisense sequence(5'to3')** | **Size(bp)** |
| --- | --- | --- | --- |
| Kappa | TGAGCAAAGCAGACTACGAGA | GGGGTGAGGTGAAAGATGAG | 231 |
| E1AF | ACAGGAACAGACGGACTTCG | CACCAGAAATTGCCACAGC | 265 |
| Elf-1 | TCAAGTCCAGGGGTAAAAGG | TTCATCCTGCATGGTACTGG | 231 |
| Elk-1 | ATTCCACCTTCACCATCCAG | AACCCGGCTCCACATTAAG | 243 |
| ER71 | CGTTTGCTCCGAACCGAGC | GCTTTCTCTTGCGCTCGCC | 218 |
| ERF | CCTGCGCTATTACTATAACAAGCG | CACCTCGGAGGGCGTTGAG | 205 |
| ERM | CCACCTCCAACCAAGATCAAACG | CACCTTGAACTGGGCCAGCTG | 268 |
| ESE1 | GCAACTACTTCAGTGCGATGTAC | CATGTCACATCGTGAGAAGTCAATG | 257 |
| GABPα | CACCATGCTGAATCAGAAGC | TGCTGAATTCCTTCATTACCC | 235 |
| MEF | GCAGCACCATCTATCTGTGG | ACTGGTACACCAGCCTCTGC | 252 |
| NERF | ACCACTGCATCTGTGTCAGC | TGCATGGTGATTTTGTCTCC | 242 |
| NET | TCCACTGCTCTCCAGCATAC | AATTGTGGCCAGACGTCATC | 211 |
| PDEF | GCTCAAGGACATCGAGACG | TGAAGTCCGCTCTTTCATCC | 271 |
| PE1 | GCAGCAGGGAGAGTACGG | GACCGAATGTTGATGAATGG | 228 |
| Sap-1 | GCAGAAGCCTCAGAACAAGC | TTGGATCCATGTTCAAAATCTCTGG | 242 |
| TEL | CCATCAACCTCTCTCATCGG | GGCTCTGGACATTTTCTCATAGG | 284 |
| TEL2 | AGGGCTTACCAGCAACTTCG | GGCTCATATCGGGTATCAAGG | 228,273 |
| PU.1 | CCACTGGAGGTGTCTGACG | GTCATCTTCTTGCGGTTGC | 248 |
| Spi-B | AGAACTTCGCTAGCCAGACC | ACGCACTCACGCATGTCC | 237,241 |
| Ets-1 | ACCCAGCCTATCCAGAATCC | TCTGCAAGGTGTCTGTCTGG | 225 |
| Ets-2 | CAGTTATACCTGCAGCTGTGC | TTGTGGATGATGTTCTTGTCG | 256 |
| LMP1 | CGTTATGAGTGACTGGACTGGA | TGAACAGCACAATTCCAAGG | 221 |
| Actin | TTCCAGCCTTCCTTCCTGGG | TTGCGCTCAGGAGGAGCAAT | 224 |
| GAPDH | CAAAGTTGTCATGGATGACC | CCATGGAGAAGGCTGGGG | 195 |
